# Medication-related osteonecrosis of the jaw: a preliminary retrospective study of 130 patients with multiple myeloma

**DOI:** 10.1186/s40902-016-0099-4

**Published:** 2017-01-05

**Authors:** Woo-Sung Choi, Jae-Il Lee, Hyun-Joong Yoon, Chang-Ki Min, Sang-Hwa Lee

**Affiliations:** 1 Department of Oral and Maxillofacial Surgery, College of Medicine, Soonchunhyang University Cheonan Hospital, Cheonan, Republic of Korea; 2 Department of Oral and Maxillofacial Surgery, Seoul St. Mary’s Hospital, College of Medicine, The Catholic University of Korea, Seoul, Republic of Korea; 3 Department of Oral and Maxillofacial Surgery, College of Medicine, Bucheon St. Mary’s Hospital, College of Medicine, The Catholic University of Korea, Bucheon, Republic of Korea; 4 Department of Hematology, Seoul St. Mary’s Hospital, College of Medicine, The Catholic University of Korea, Seoul, Republic of Korea; 5 Department of Dentistry, St. Paul’s Hospital, College of Medicine The Catholic University of Korea, 180 Wangsan-ro, Dongdaemun-gu, Seoul 130-709 Republic of Korea

**Keywords:** Multiple myeloma, Bisphosphonate, Medication-related necrosis of the jaw

## Abstract

**Background:**

Multiple myeloma (MM) is characterized by a neoplastic proliferation of plasma cells primarily in the bone marrow. Bisphosphonates (BP) are used as supportive therapy in the management of MM. This study aimed to analyze the incidence, risk factors, and clinical outcomes of medication-related necrosis of the jaw (MRONJ) in MM patients.

**Methods:**

One hundred thirty MM patients who had previous dental evaluations were retrospectively reviewed. Based on several findings, we applied the staging and treatment strategies on MRONJ. We analyzed gender, age, type of BP, incidence, and local etiological factors and assessed the relationship between these factors and the clinical findings at the first oral examination.

**Results:**

MRONJ was found in nine male patients (6.9%). The mean patient age was 62.2 years. The median BP administration time was 19 months. Seven patients were treated with a combination of IV zoledronate and pamidronate, and two patients received single-agent therapy. The lesions were predominantly located in the mandible (*n* = 8), and the most common predisposing dental factor was a history of prior extraction (*n* = 6). Half of the MRONJ were related to diseases found on the initial dental screen. Patients with MRONJ were treated with infection control and antibiotic therapy. When comparing between the MRONJ stage and each factor (sign, location, etiologic factor, BP type, treatment, and outcome), there were no significant differences between stages, except for between the stage and sign (with or without purulence).

**Conclusions:**

For prevention of MRONJ, we recommend routine dental examinations and treatment prior to starting BP therapy.

## Background

Multiple myeloma (MM) is characterized by a neoplastic proliferation of plasma cells mostly within the bone marrow [1]. MM comprises 0.5% of all cancers in Korea [2]. Worldwide annual incidence is 1.5 per 100,000 individuals. Anemia, renal dysfunction, infections, and bone lesions are the most common complications of MM. In the majority of patients, slow and steady progressive bone damage, or osteolytic lesions, may lead to fractures of the long bones or compression fractures in the spine. Bone pain is often a symptom of this disease, especially severe back pain. Bisphosphonates (BPs) are used in the management of MM as supportive therapy to inhibit the progression of osteoclast activity, which reduces skeletal-related morbidity and mortality [3].

BPs are non-metabolized pyrophosphate analogues that are capable of localizing in bone and inhibiting osteoclast function [4]. These drugs act at the site of active bone remodeling by binding to hydroxyapatite, inhibiting osteoclast development and migratory activity. Inhibiting osteoclast function leads to cell death, which decreases bone resorption without affecting bone mineralization [5]. These non-metabolized analogues are maintained at high concentrations in bone resorption lacunae for an extended period, allowing long-term inhibition of osteoclastic function. In addition, BPs can inhibit bone resorption and decrease bone turnover at the tissue level, as assessed by biochemical markers [4].

In addition to oral BPs used for osteoporosis and osteogenesis imperfecta, intravenous BPs are effective in the treatment and management of several conditions. Intravenous (IV) BPs treat cancer-related conditions, including hypercalcemia of malignancy, lytic lesions in MM, and the effects of bone metastasis in the context of solid tumors, such as breast cancer, prostate cancer, and lung cancer. In metastatic disease to the bone, BPs prevent skeletal complications, reduce bone pain, and improve the quality of life. Additionally, there is some evidence that BPs also have anti-tumor activity. Bisphosphonate therapy is recommended for all patients with multiple myeloma requiring chemotherapy, whether bone lesions are evident or not [6].

Adverse effects associated with the use of BP include pyrexia, gastrointestinal symptoms, hypocalcemia, and renal dysfunction [3, 7]. In 2003, Marx [8] described bisphosphonate-related osteonecrosis of the jaw (BRONJ) as a new complication associated with nitrogen-containing BP use. The American Association of Oral and Maxillofacial Surgeons (AAOMS) published a position paper providing guidance regarding the prevention, diagnosis, and treatment of BRONJ according to the different stages. BRONJ is diagnosed when BP administration is followed by bone exposure that does not heal within 8 weeks of identification and when patients have no history of local radiation therapy [9, 10]. Recently, the term medication-related osteonecrosis of the jaw (MRONJ) was introduced in the updated AAOMS position paper discussing bisphosphonate-related osteonecrosis of the jaw. This updated paper accommodates the growing number of osteonecrosis cases involving the maxilla and mandible associated with other anti-resorptive (denosumab) and anti-angiogenic therapies.

Presently, few reports addressing the epidemiology of MRONJ in patients with MM have been published. Furthermore, few studies with stage and treatment strategies that are recommended by the AAOMS have been published yet. The aim of this study was to analyze the incidence of MRONJ in multiple myeloma patients and to analyze the systemic and local risk factors, including stage and clinical outcome, with standard treatments recommended by the AAOMS position paper. This report focuses on both medical and dental databases at a single-center.

## Methods

This retrospective study included MM patients with a history of intravenous bisphosphonate therapy at the Department of Hematology, St. Mary’s Hospital of the College of Medicine, The Catholic University of Korea, who had been referred to the Department of Dentistry for oral examination or dental care. MRONJ was defined when all of the following characteristics were present: (1) current or previous treatment with BPs, (2) exposed bone in the maxillofacial region persisting for more than 8 weeks, and (3) no history of radiation to the jaw. We recorded patients’ clinical signs and symptoms, including the location of exposed and necrotic bone, evidence of infection, pain, and the extent of osteolysis. Based on these findings, we applied the staging and treatment strategies described by the AAOMS position paper on MRONJ to each patient (Table 1) [10]. We analyzed gender, age, type of BP, incidence, and local etiological factors, including their relationship to the clinical findings at the first oral examination. The institutional review board of St. Mary’s Hospital approved this study.

**Table 1:** Staging and treatment strategies described by the American Association of Oral and Maxillofacial Surgeons position paper on MRONJ of the jaw

MRONJ staging	Description	Treatment strategies
At risk category	No apparent necrotic bone in patients who have been treated with either oral or IV bisphosphonates	No treatment indicated Patient education
Stage 0	No clinical evidence of necrotic bone but non-specific clinical findings and symptoms	Systemic management, including use of pain medication and antibiotics
Stage 1	Exposed and necrotic bone in patients who are asymptomatic and have no evidence of infection	Antibacterial mouth rinse. Clinical follow-up on a quarterly basis. Patient education and review of indications for continued bisphosphonate therapy.
Stage 2	Exposed and necrotic bone associated with infection as evidenced by pain and erythema in the region of the exposed bone with or without purulent drainage	Symptomatic treatment with oral antibiotics. Oral antibacterial mouth rinse. Pain control. Superficial debridement to relieve soft tissue irritation.
Stage 3	Exposed and necrotic bone in patients with pain, infection, and one or more of the following: exposed and necrotic bone extending beyond the region of alveolar bone (i.e., inferior border and ramus in the mandible, maxillary sinus and zygoma in the maxilla) resulting in pathologic fracture, extra-oral fistula, oral antral/oral nasal communication, or osteolysis extending to the inferior border of the mandible of sinus floor	Antibacterial mouth rinse. Antibiotic therapy and pain control. Surgical debridement/resection for long-term palliation of infection and pain.

For statistical analysis, SAS software package (Version 9.3, SAS Institute) was used. Categorical variables such as sign, location, etiologic factors, BP type, treatment, and outcome were analyzed by Fisher’s exact tests, and continuous variables such as duration were analyzed by Wilcoxon tests. A *p* value <0.05 was considered statistically significant.

## Results

**Fig. 1:**
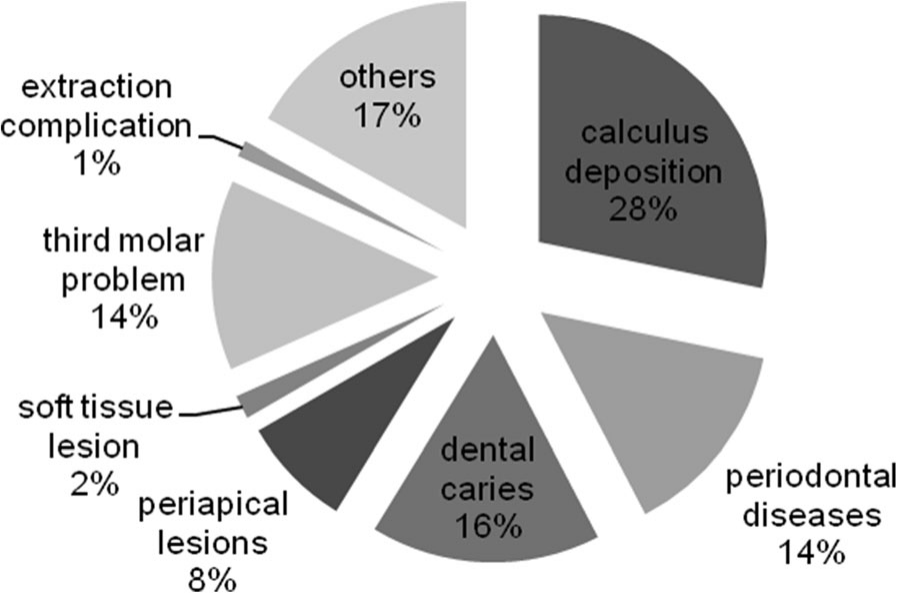
Distribution of the dental problems in multiple myeloma patients at their initial visit

**Fig. 2:**
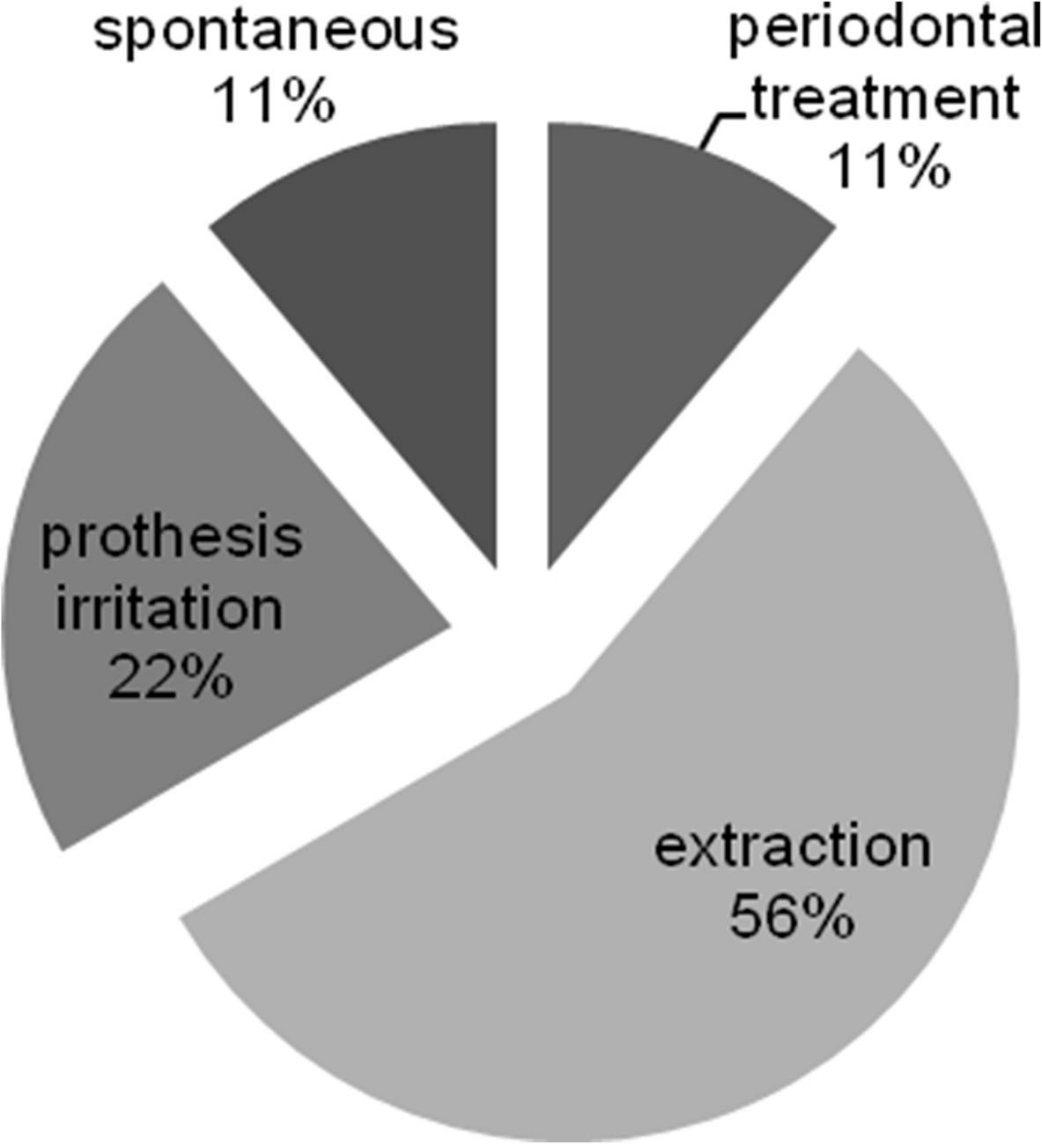
Distribution of cases according to the preceding events

**Fig. 3:**
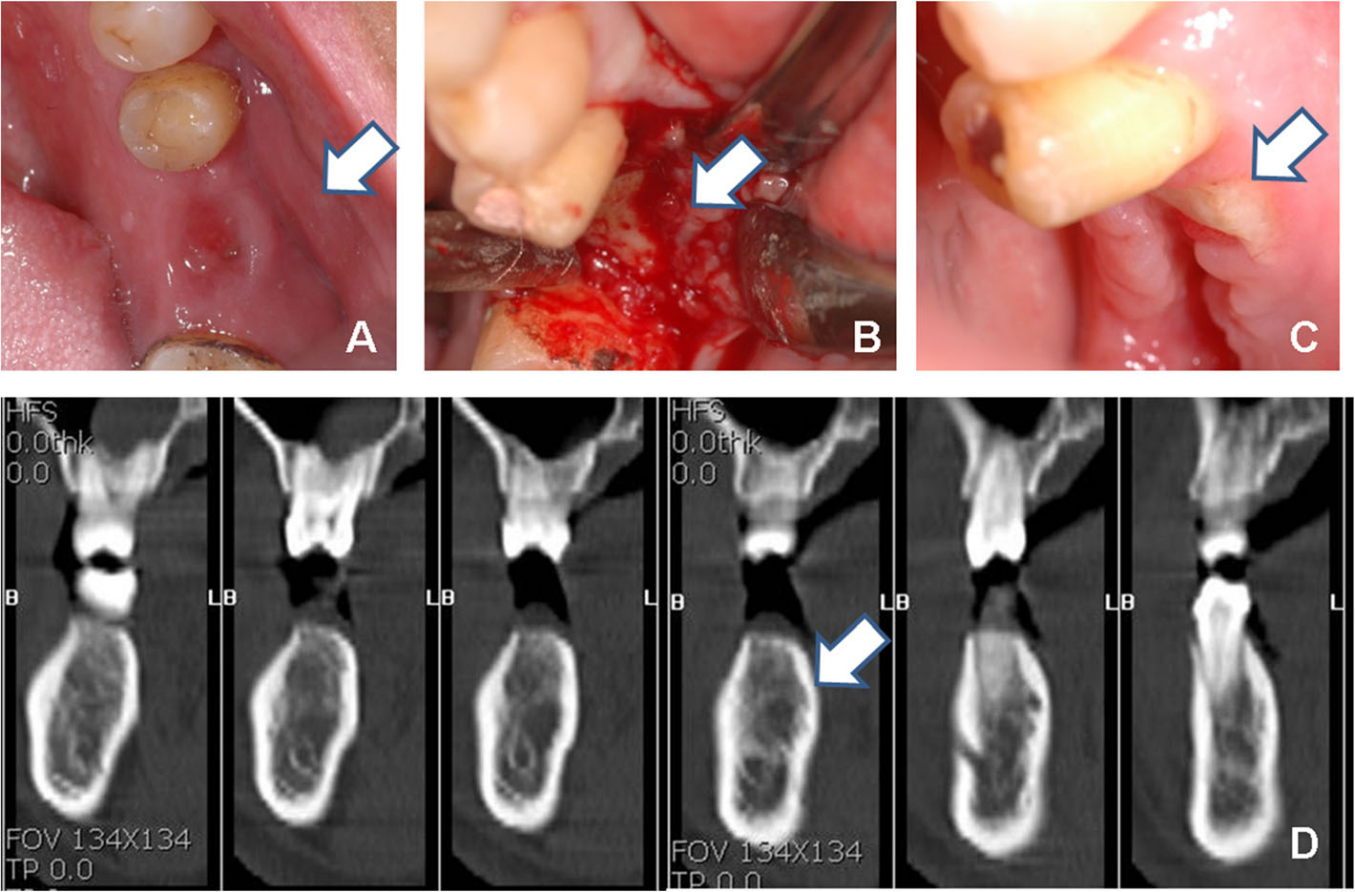
Clinical features and CT of MRONJ patient. **a** Mucosal fistula on the pontic area. **b** Intraoperative findings during bone biopsy. **c** Exposed alveolar bone. **d** CT findings

A total of 130 MM patients visited the Department of Dentistry. There were 57 females and 74 males. The mean patient age was 57 years (range 36–76 years). At the initial dental examination, we found 50 cases with calculus deposition, 29 cases with dental caries, 25 cases with periodontal diseases, 24 cases with third molar disease, 14 cases with periapical lesions, and 30 cases with other diseases, including hypersensitivity, cervical abrasion, and attrition. Only two cases of MRONJ associated with tooth extraction were present. In addition, we saw 15 MM cases without dental disease (Fig. 1). However, seven of these patients later developed MRONJ. Overall, jaw necrosis occurred after bisphosphonate therapy in nine cases (6.9%). These patients were all male, and the mean patient age was 62.2 years. The median length of BP exposure was 19 months (range 8–69 months). All patients received intravenous BP therapy. Seven of the patients were treated with a combination of zoledronate and pamidronate, one patient received zoledronate alone, and one received pamidronate alone. Four of the patients were also given an oral bisphosphonate (panonin) (Table 2). The observed lesions were predominantly located in the mandible. Only one case had a maxillary lesion. At the initial visit, only two of the patients with MRONJ presented with exposed bone at the extraction socket. In the remaining patients, we found periodontal diseases (*n* = 5), calculus deposition (*n* = 2), mucosal irritation (*n* = 1), third molar problems (*n* = 1), and periapical lesions (*n* = 2). Half of the MRONJ cases occurred at sites that were related to the diseases seen at the initial visit. The most common predisposing dental factor was dental extraction (*n* = 6), followed by prosthesis irritation (*n* = 2), and periodontal therapy (*n* = 1) (Fig. 2). However, one patient developed MRONJ spontaneously (Fig. 3).

**Table 2:** Clinical characteristics of MM patients with MRONJ

No.	Stage (sign)	locLocation	Etiologic factor	BP types (duration in months)	Treatment	Outcomes (stage)
1	II (Bone exposure, pus discharge, pain)	Mandible molar	Denture irritation	Zoledronate, pamidronate (19)	I&D, antibiotics, dressing, biopsy	Bone exposure (stage I)
2	II (Bone exposure, pus discharge, pain)	Mandible molar	Pontic irritation	Zoledronate, pamidronate (19)	Antibiotics, dressing, biopsy	Bone exposure (stage I)
3	II (Bone exposure, pus discharge, pain)	Mandible molar	Tooth extraction	Zoledronate (15)	I&D, antibiotics, dressing	Bone exposure (stage I)
4	II (Bone exposure, pain)	Mandible molar	Tooth extraction	Zoledronate, Pamidronate (69)	Antibiotics, Dressing	Bone exposure (stage I)
5	II (Bone exposure, pus discharge, pain)	Mandible molar	Tooth extraction	Zoledronate, pamidronate (11)	Saucerization I&D, antibiotics, dressing	No follow-up
6	I (Bone exposure)	Mandible molar	Spontaneous	Zoledronate, pamidronate (25)	Antibiotics, dressing	Bone exposure (stage I)
7	II (Bone exposure, pus discharge, pain)	Maxilla molar	Gingival curettage	Zoledronate, pamidronate (40)	Antibiotics, dressing	Bone exposure with pain (stage II)
8	III (Pain, Osteolytic lesion on Mn. Basal bone)	Mandible molar	Tooth extraction	Pamidronate (8)	Antibiotics, dressing	No f/u
9	I (Bone exposure)	Mandible molar	Tooth extraction	Zoledronate, pamidronate (17)	Antibiotics, dressing, biopsy	Bone exposure state (died due to systemic disease)

According to the AAOMS staging system, five cases were stage II, two cases were stage I, and one case was stage III. One patient progressed from stage 0 to stage II after the biopsy. This patient showed a fistula of unknown origin under the bridge pontic. The Bone scan revealed intense hot uptake in the mandible in this patient, and CT and biopsy exhibited chronic osteomyelitis likely secondary to actinomycosis. However, a few days after the bone biopsy, the bone became exposed despite repair with a primary suture (Fig. 1).

The main treatment methods for patients with MRONJ focus on infection control and antibiotic therapy as recommended by the AAOMS. One patient with stage I MRONJ died after the treatment due to underlying disease, and two patients were lost to follow-up. All six remaining patients presented with non-healing exposed bone during the follow-up. Five of the six patients were stage I, and the remaining patient was stage II. Notably, this stage II patient did not improve with treatment (Table 2).

When comparing between stage and each factor (sign, location, etiologic factor, BP type, treatment, and outcome), there were no significant differences between groups, except for between the stage and sign (with or without pus) (Table 3).

**Table 3:** Comparing patients with MRONJ between MM stages according to each factor (sign, location, etiologic factor, BP type, treatment, and outcome) of MM

	Stage			
	1	2	3	*P* value
Number of patients	2	6	1	
Location (%)				1
Mandible	2 (100)	5 (83.33)	1 (100)	
Maxilla	0 (0)	1 (16.67)	0 (0)	
Infection (%)				0.0476*
Yes	0 (0)	5 (83.33)	0 (0)	
No	2 (100)	1 (16.67)	1 (100)	
Etiology (%)				0.7222
Extraction	1 (50)	3 (50)	1 (100)	
Prosthesis problem	0 (0)	2 (33.33)	0 (0)	
Periodontal problem	0 (0)	1 (16.67)	0 (0)	
Spontaneous	1 (50)	0 (0)	0 (0)	
Bisphosphonate type (%)				0.25
Combination^a^	2 (100)	5 (83.33)	0 (0)	
Only zoledronate	0 (0)	1 (16.67)	0 (0)	
Only pamidronate	0 (0)	0 (0)	1 (100)	
Treatment^b^ (%)				0.6429
Conservative	2 (100)	3 (50)	1 (100)	
Invasive	0 (0)	3 (50)	0 (0)	
Outcome (%)				1
1	1 (100)	4 (80)	0 (0)	
2	0 (0)	1 (20)	0 (0)	
Duration in months	21 (17–25)	19 (11–69)	9 (9–9)	0.2992

Categorical variables such as sign, location, etiologic factor, BP type, treatment, and outcome were analyzed by Fisher’s exact test. Continuous variables such as duration were analyzed by Wilcoxon test

^*^Statistically significant (*p* value <0.05)

^a^Combination of zoledronate and pamidronate

^b^Conservative: antibiotic therapy and dressing invasive: biopsy or saucerization

## Discussion

The exact mechanism of action underlying the pathogenesis of MRONJ is uncertain. MRONJ may be associated with medication-related apoptosis of osteoclasts or to the anti-angiogenic and suppressive effects that BPs have on endothelial cells [11]. The strong association of intravenous nitrogen-containing BPs with MRONJ compared to oral BPs suggests that intravenous BPs are more bioavailable, allowing increased incorporation into the bone matrix. Only 1% of oral BPs are circulated in the serum due to delayed gastrointestinal tract absorption.

The prevalence of MRONJ in patients treated with intravenous BPs ranges from 0.8 to 12% [10]. According to a cohort study, the estimated incidence of MRONJ in MM patients is 8.42%, and the highest incidence is in patients who received a combination of pamidronate and zoledronate (range 5 to 51%). Zoledronate is associated with MRONJ in 3 to 11% of MM cases, while the pamidronate-related frequency of MRONJ ranges from 0 to 18% [3]. The overall incidence of MRONJ in our study was 6.9% (9/130 MM patients). Of these nine patients, 77.7% (*n* = 7) received a combination of pamidronate and zoledronate.

Studies evaluating the duration of bisphosphonate therapy as a risk factor for MRONJ development in MM patients have yielded inconsistent results [6, 9]. In our patients, the median length of BP exposure was 19 months, which is similar to the results of Berenson et al. (18 months of zoledronate) [12]. However, our results are not consistent with those of Corso et al. [13] (47 months of pamidronate and zoledronate combination therapy), Zervas et al. [14] (24 months of pamidronate), and Dimopoulos et al. (53.4 months of pamidronate and zoledronate combination therapy) [15].

It is thought that the jaw is more predisposed to MRONJ compared to other bones. This may be due to healing of open bone wounds, vulnerability to bacterial infections, and that BPs are preferentially deposited in bone with higher turnover rates, such as the jaw [16, 17]. Wen et al. [18] supported the theory that BPs preferentially deposit into bones with higher rates of remodeling, such as the jaw, relative to non-oral sites. Using athymic rats, they demonstrated a significantly higher release of hydroxyapatite-bound bisphosphonate in oral bones compared to axial and appendicular sites. After injection of fluorescent pamidronate into the mice, more signal was detected and was retained for longer time periods in the mandible than in the femur [19], which demonstrates that the mandible has a high affinity for BPs. Notably, MRONJ is twice as common in the mandible as in the maxilla [10]. Marx et al. [20] reported that 68.1% of the MRONJ cases occur in the mandible and 27.7% occur in the maxilla. Similar results were found in a study by Bardos et al. [5], where 15 of 22 MM patients with MRONJ showed mandibular lesions, five showed both mandibular and maxillary lesions, and two were in the maxilla only. In our study, only one patient presented with MRONJ in the maxilla. One possible explanation for osteonecrosis secondary to BP use, especially in the mandible, may be BP’s anti-angiogenic effects, or the anatomic and physiologic features of the mandible might increase the risk of MRONJ [21, 22]. Unfortunately, the exact mechanism underlying the higher incidence of MRONJ in the mandible remains unknown.

In MRONJ, dental extraction is the biggest local risk factor [6]. The surgical damage to the jaw that occurs after extraction, especially the damage to the alveolar bone with open bone wounds, seems to be the most potent trigger for MRONJ. In extractions, jaw bone remodeling is depressed, leading to the spontaneous breakdown of the wound after the tooth is removed. Notably, the site of the pre-existing bone might not be of prime importance. Woven bone is not altered by BPs, but remodeling of woven bone into lamellar bone might be impaired, leading to MRONJ [23]. Patients receiving intravenous BPs and undergoing dentoalveolar treatment are at least seven times more likely to develop MRONJ than those who are not [5, 10]. Tooth extraction is associated with 77% of MRONJ cases in the patients with intravenously administrated BPs [6]. In our study, the most common risk factor for MRONJ was dental extraction (*n* = 6), followed by prosthesis irritation (*n* = 2), periodontal therapy (*n* = 1), and unknown causes (*n* = 1). Half of the MRONJ locations coincided with the site of the dental problem seen at the first visit.

This retrospective study has some limitations. Firstly, we did not evaluate the patients’ oral health prior to initiating bisphosphonate therapy. However, we found that periodontal disease, including calculus deposition, was the most frequent dental disease in the MM patients at the initial dental examination such as in them with MRONJ. Aside from the two patients who initially presented with MRONJ, the majority of MM patients had periodontal problems, such as calculus deposition (*n* = 50), periodontal lesions (*n* = 25), and dental caries (*n* = 29). These results are similar to those of MM patients with MRONJ. In MM with MRONJ, the most common dental disease was periodontal disease (*n* = 5), followed by calculus deposition (*n* = 2), periapical lesions (*n* = 2), mucosal irritation (*n* = 1), and third molar problems (*n* = 1). Thus, clinical examinations, including panoramic radiography, may help detect dental problems and improve oral health before and during bisphosphonate therapy.

Based on our data, prevention of MRONJ is optimal because its management is quite challenging. While some case reports have demonstrated surgical treatment with good outcomes, generally, aggressive surgery has been counterproductive, often exacerbating the bone exposure [24]. Hence, most publicized studies suggest that initial treatment (stage I, II) should be conservative, focusing on systematic antibiotic therapy, irrigation to optimize oral hygiene, and careful removal of sequesters [10, 17, 25]. However, only 23–53% of all patients achieve resolution of mucosal discontinuities [26, 27]. Bamias et al. [7] demonstrated that antibiotic therapy resulted in temporary improvement, but only one patient showed sustained improvement of their osteonecrosis after multiple courses of antibiotics. The remaining 11 MM patients with MRONJ had persistent disability, mainly experiencing recurrence with purulent discharge and pain after discontinuing their antibiotics. These results coincide well with ours. In this present study, after conservative treatment as recommended by the AAOMS, the majority of stage II patients has been improved and was reclassified as stage I, but all patients remained with unhealed exposed bone at the follow-up appointment.

## Conclusions

Osteonecrosis of the jaw has been recognized as a serious complication of intravenous BP treatment, especially in patients with MM. We found that the majority of MM patients with MRONJ received a combination of pamidronate and zoledronate. The mandible, particularly at molar extraction sites, was the most frequent area affected. In some patients, the location of MRONJ and the type of dental problem coincided with the oral condition seen at the initial visit. Management regimens that lead to complete resolution of MRONJ remain elusive. While conservative therapy improved some conditions, all patients were left with some remaining exposed bone. Therefore, the prevention of MRONJ is crucial. Routine dental examinations and treatment of dental diseases should be performed prior to initiating bisphosphonate therapy.
